# FRailty and Arterial stiffness – the role of oXidative stress and Inflammation (FRAXI study)

**DOI:** 10.1177/11772719221130719

**Published:** 2022-10-18

**Authors:** Ekow Mensah, Khalid Ali, Winston Banya, Frances Ann Kirkham, Manuela Mengozzi, Pietro Ghezzi, Chakravarthi Rajkumar

**Affiliations:** 1 Brighton and Sussex Clinical Trials Unit, University Hospitals Sussex NHS Trust, Brighton, UK; 2 Department of Medicine, Brighton and Sussex Medical School, University of Sussex, Brighton, UK; 3 Research Office, Royal Brompton and Harefield Clinical Group, Guy’s and St. Thomas’ NHS Foundation Trust, London, UK; 4 Università degli Studi di Urbino, Italy

**Keywords:** Frailty, older adults, inflammation, inflammaging, oxidative stress, vascular stiffness, vascular ageing, nitrotyrosine, 8-hydroxy-2’-deoxyguanosin, c-reactive protein, interlukin-6, biomarkers

## Abstract

**Objective:**

There is an association between frailty and arterial stiffness. However, arterial stiffness does not uniformly correlate with the spectrum of frailty states. Both oxidative stress and inflammaging contribute to vascular ageing. There are no human studies exploring links between arterial stiffness, oxidative stress, inflammaging and frailty. Our objective is to investigate arterial stiffness and inflammaging as predictors of frailty states.

**Methods:**

An observational longitudinal cohort study will be used to examine the association between arterial stiffness, oxidative stress and inflammation in 50 older adults (⩾70 years) with clinical frailty scores (CFS) ⩽6 over 6 months. All study measurements will be taken at baseline. Frailty assessment will include hand-grip strength, timed-up and go test, mini-mental state examination, geriatric depression scale and sarcopenia using body composition measurements with Tanita^®^. Arterial stiffness measurements will include carotid-femoral pulse wave velocity (cfPWV) and carotid-radial pulse wave velocity (crPWV) using Complior (Alam Medical, France). CAVI device will measure Cardio-ankle vascular index and ankle brachial index (ABI). Oxidative stress blood markers nitrotyrosine (NT) and 8-hydroxy-2’-deoxyguanosin (8-oxo-dG) and inflammation markers high-sensitive C-reactive protein (hs-CRP) and interlukin-6(IL-6) will be measured at baseline and 6 month along with lipid profile and glycated haemoglobin.

**Results (data analysis plan):**

Descriptive statistics for continuous data using means and standard deviations for normality distributed variables or medians and inter-quartile ranges for skewed variables will be used. Participants will be categorised into CFS 1-3, and CFS 4-6. Categorical data will use frequencies and comparison between groups. Change in frailty between the groups over 6 months will be compared using paired t-test. Simple linear regression will be done between frailty measures, arterial stiffness, inflammation and oxidative stress biomarkers. Significance will be at *P* < .05.

**Conclusion:**

This study data will inform a larger, multi-centre study exploring further the interplay between frailty, biomarkers and arterial stiffness parameters.

## Introduction

As the world’s population keeps ageing, a corresponding increase in older adults with frailty occur.^1^ In the UK, it is estimated that close to a fifth (18%) of the population are 65 years and over, out of which 7% are known to be severely frail.^2,3^ Indeed, the House of Lords report, considers healthy ageing as one of the 4 cross cutting ‘grand challenges’ in the UK,^4^ thus emphasising the importance of addressing matters related to ageing and frailty.

Frailty as a geriatric syndrome, is associated with an increased risk of adverse outcomes,^5,6^ such as falls, reduced mobility and independence, increased hospitalisations, cognitive decline, disability and death.^7^ It is known to be a risk factor of cardiovascular disease and when coupled with arterial stiffness, potentiates this risk.^8,9^ The association between frailty and arterial stiffness is well known.^8,10^ In a cross-sectional study involving 2171 community-dwelling participants aged 60 years and over, arterial stiffness was noted to be strongly associated with pre-frail and frail status.^11^ Although this association exists, arterial stiffness does not uniformly correlates with the various stages of frailty^12-14^ though it is considered to be the hall mark of vascular ageing.^8^

In the mechanism of vascular ageing, inflammation and oxidative stress have been observed as predominant underlying factors aside others such as genomic instability.^15-17^ Studies have shown strong association between oxidative stress and inflammation with frailty.^18-21^ Two oxidative biomarkers malondialdehyde (MDA) and protein carbonylation were noted to have strong association with frail elderly people in 742 adults studied, compared to the pre-frail and non-frail.^22^ In a prevalent-case control study of 55 adults greater than 65 years, compared to healthy controls, frail adults had a higher percentage of circulating 8-isoprostane (*P* = .001), a validated oxidative stress marker.^23^ A significant increase in 8-hydroxy-2’-deoxyguanosine (8-OHdG) and 8-isoprostane levels, together with higher levels of interleukin-6, were noted to be present in the prefrail and frail elderly adults in contrast to the non-frail in a study of 140 patients with Alzheimer’s.^24^ Similarly, another study in 2019 examining the relationship between inflammation and frailty among 817 adults 65 years and older, showed 2 inflammatory markers (Erythrocyte sedimentation rate-ESR and Neutrophil to lymphocyte ratio-NLR) to be significantly elevated in the frail group compared to the non-frail groups.^25^ In a systematic review with meta-analysis on the association of inflammatory biomarkers and frailty, there was a strong association between these markers (Interlukin-6 – IL-6, and C-reactive protein – CRP) and frailty status (pre-frail and frail), a proof of the role of inflammation in frailty.^26^

Although oxidative stress and inflammaging (the chronic state of inflammation in ageing) are known to be associated with frailty and considered to be underlying vascular ageing, there are no observational studies in human, on the effects of these 3 on frailty. Frailty is thought to be potentially reversible especially in its early stages.^27-34^ Therefore, its early detection and management is key to prevent and reduce its impact on older adults.^26,35,36^ Thus, a major rationale for this pilot study is to be able to offer an objective measure based on arterial stiffness for frailty, incorporating the roles oxidative stress and inflammation play.

This would help ultimately in the early detection of frailty, and subsequently contribute to the body of evidence-based medicine where clinicians may in the medium-to-long term, use these bio-markers to help assess degrees of frailty, and aid in slowing down progression of frailty, by addressing issues of inflammation and oxidative stress.

### Study aim

To study the mediating roles of oxidative stress, inflammation and arterial stiffness in the pathogenesis of frailty.

### Study objective

To undertake a pilot study exploring the use of markers of inflammation, oxidative stress and arterial stiffness as an objective measure for frailty.

### Hypotheses

Frail older adults with arterial stiffness have a corresponding increase in biomarkers of oxidative stress and inflammation.

Research Question:

Is oxidative stress and inflammation associated with arterial stiffness in frail older people?

## Study Design

This is an observational longitudinal cohort study looking at the association between oxidative stress and inflammation among older adults with varying frailty status and arterial stiffness over a period of 6 months. The main goal of this pilot study is to provide preliminary data to inform a follow-up larger, national, multi-centre study exploring further the links between frailty and vascular parameters.

## Study Methods: Participants, Interventions and Outcomes

### Study setting

The study will be undertaken at the University Hospitals Sussex NHS Foundation Trust, Clinical Research Facility (CRF). Participants will be invited for recruitment from outpatients at University Hospitals Sussex NHS Trust.

### Eligibility criteria

Inclusion criteria:

1. Age 70 years and above with a Clinical Frailty score ⩽6/Electronic Frailty Index ⩽0.36.
2. Patients who have capacity to give informed consent.

Exclusion criteria:

1. Malignancy, with current active treatment
2. Patient unable to give informed consent
3. Patient with a Rockwood Frailty score 7 to 9 or Electronic Frailty Index in the severe frailty group (>0.36).

Should a participant loose capacity during the study, they will be excluded from further involvement in the study, however the data collected up until the time of loss of capacity will be used. Initially, capacity to participate in the study will be assessed prior to consent by a member of the research team, and again by the research team member over the phone prior to the study visit.

### Measurements

At recruitment and at follow up, frailty status of participants would be assessed using the Rockwood/Clinical Frailty score (CFS) and the electronic Frailty index (eFI) score. The Rockwood/CFS is a frailty assessment spectrum grouped from 1 (very fit) to 9 (terminally ill). The electronic frailty index (eFI) classifies frailty into 4 groups spanning from fit (eFI < 0.12) to severely frail (eFI > 0.36). Participants in this study, would be categorised based on their scores as no-mild frailty (CFS ⩽ 3, eFI ⩽ 0.24) and moderate frailty (CFS 4-6, eFI 0.24-0.36). The choice of stratifying the study participants into a spectrum of frailty states from not-frail to mildly frail and moderately frail is based on the knowledge that progression towards advanced frailty states can be delayed and/ or reversed if the right intervention is implemented.^31,33,37^

Arterial stiffness will be measured in 2 ways: using Complior^®38^ and CAVI^®.39^ Assessment of arterial stiffness will be done at room temperature with the patient supine and after having rested for 10 minutes. Patients will be asked to refrain from smoking, eating or drinking caffeinated drinks for 3 hours before measurements and to avoid consuming alcohol for 10 hours before measurements. Participants will be offered reimbursement for travel expenses.

(a) The Complior^®^ is a non-invasive device which measures Pulse Wave Velocity and central pressures. The carotid, femoral and radial pulses are palpated and marked, and measurements are taken between them. The wave forms are then measured and recorded on the Complior^®^ programme using small sensors. We will perform at least 2 measurements, and if similar will use the mean of these. If these differ by more than 0.5 m/s, a third measurement will be performed. The PWV value should be the median of these measurements.

(b) Cardio-Ankle Vascular Index (CAVI^®^) is another index which assesses arterial stiffness, this time it uses measurements in all 4 limbs. CAVI^®^ then equates the stiffness using the pulse wave between the heart and the ankles. Complior and CAVI measurements will be done by the same person to ensure consistency. These 2 measurements will be done only once at baseline and not at follow up since we do not expect any changes within 6 months.

(c) A blood sample will be obtained by a trained research nurse or doctor. The participant will be asked to give approximately 20 mls of blood (one and a half tablespoons), plasma samples will then be stored at −80°C until they are tested. All participants will have samples taken to measure markers of oxidative stress and inflammation in the clinical research laboratory at the University of Sussex. Inflammatory markers to be measured would include high sensitivity C-reactive protein (hs-CRP) and Interlukin-6 (IL-6). Markers for oxidative stress to be measured would include nitrotyrosine (NT) and 8-hydroxy-2’-deoxyguanosin (8-oxo-dG). Inflammation and oxidation profiles will be measured by ELISA using commercially available assay kits. Samples would be handled by the research nurse or doctor for storage before being transported to the research laboratory at the University of Sussex to be assayed together.

(d) Other biochemistry measurements would be recorded from the hospital computer system which would include lipid profile, thyroid function and full blood count. Other routine clinical observations to be checked would be blood pressure, body mass index (BMI) and random blood sugar (RBS).

(e) At recruitment and follow up, cognition of participants would be assessed using the Abbreviated Mental Test Score (AMTS) and Montreal Cognitive Assessment (MoCA). These readings would be taken from the patients’ file. Patients with scores less than 8 on the AMTS and 23 on MOCA will be excluded from the study.

(f) Measures of frailty to be assessed will include hand grip strength using the hand dynamometer, timed up and go test (TUGT) and body impedance with the Tanita.

(g) Participants will be followed up at 6 months for second measurements to be taken. Prior to follow up visit, participants would be contacted via telephone call for booking of appointment. All measurements taken during baseline/recruitment would be repeated except for the arterial stiffness. Snacks will be provided for the participants after their participation both at recruitment and follow up and their transportation bill catered for.

### Sample size

This is a pilot study with no formal sample size calculation done. We aim to recruit 40 participants who fit the eligibility criteria, but this number will be adjusted upwards to 50 participants to account for an expected dropout rate of 20%. We aim to recruit at least 2 participants per week within 5 months. The selected sample size is considered reasonable for a pilot/feasibility study by NIHR.^40,41^ Similarly, this is in line with Browne’s rule of thumb for pilot study sample size calculations which recommends at least 30 subjects for pilot studies to estimate differences between 2 groups.^42^ It is further supported by Julious^43^ who suggests that a minimum of 12 subjects per treatment arm /group is relevant to reduce imprecision around the estimates of a standard deviation. The chosen sample size of 50 is a pragmatic approach considering previous studies which used similar numbers, where 28 frail older adults were compared with 27 robust non-frail older adults assessing the differences in oxidative stress markers between both groups.^23^ Participants would be stratified into 2 groups based on their frailty status as group 1 having no-mild frailty (ie, having a Rockwood frailty score ⩽3) and group 2 made up of moderate frailty with a Rockwood frailty score >3 in line with the study’s inclusion criteria. We would aim to have an equal number of 25 participants in each group.

### Recruitment and obtaining consent

Potential participants for the study will be highlighted to the research team by clinical staff within the hospital. Most of these potential participants will be identified on the care of the elderly wards and clinics. The potential participants will be invited to listen to an initial explanation of the study and asked if they would like to be considered as a participant, at this stage a participant information sheet (PIS) will be provided. This will happen whilst the potential participant is in hospital. Interested individuals will be advised to spend at least 24 hours considering involvement in the study. Following at least 24 hours, interested individuals will discuss the study again and any questions will be answered by a trained member of the research team. If the person wishes to be involved, an appointment will be made at which time formal written consent will be given prior to assessment. An appointment will be made for the follow up visit in 6 months post discharge. Participants transport fares would be catered for by the team.

Should a participant loose capacity during the study, they will be excluded from further involvement in the study, however the data collected up until the time of loss of capacity will be used.

## Data Collection, Management and Analysis

### Assessment and collection of outcome, baseline and other study data

The research staff performing assessments of arterial stiffness will be trained and validated in the approved methods of using the various pieces of equipment. The following machines are being used to assess arterial stiffness and the corresponding links to information on validity and reliability are provided.

Complior^®^: http://www.complior.com/info-center^44^
CAVI^®^: http://www.fukuda.co.jp/english/products/special_features/vasera/cavi.html^45^

Participants will be followed up at 6 months following the initial study visit. Prior to follow up visit, research staff will check if the participant is currently in hospital or if the participant is deceased. In the unlikely event that the participant has died or that they are currently an inpatient, the follow-up telephone call will not be made. Participants will be asked to provide an email address if they would like to be contacted via email to arrange the timing of the follow-up visit.

### Data entry, coding, security and storage

Data will be reviewed quarterly and at mid-point through the study to check for normality and ranges of data values. The data collected will be entered into a computer database by a nurse or doctor in the research team and will be checked by supervising members of the team to ensure high data quality.

The data collected for each participant in the study will be recorded in a case report form (CrF). Each CrF will be identified by the study number of the subject concerned. Each participant will be assigned a study number in sequential order (eg,: FRAXIS 001 through to FRAXIS 050). All information obtained during the study (with the exception of data given in the informed consent form) shall be entered into a password-protected computer database in compliance with the Data Protection Act (2018) and stored on a secured University approved server.

### Statistical analysis plan

Results will be imported into STATA for data cleaning and statistical analysis. Since this is a pilot study, analysis will be mainly descriptive. Descriptive statistics for continuous data will be assessed using means and standard deviations for normally distributed variables, or medians and interquartile ranges for skewed continuous variables. Analysis would involve looking at missing data, drop- out rate which would inform sample size calculation for a future larger study. Categorical data will be summarised using frequencies and percentages and comparison between the 2 groups (moderate frailty and no-mild frailty) done using the chi-squared or Fishers exact test.

Where data are not normally distributed these will be log transformed and presented as geometric means with their 95% confidence interval. Where data are not normally distributed even after transformation, these will be presented as median (IQR) and comparisons between groups done using the Wilcoxon rank-sum test.

The Primary outcome (change in frailty status) will be compared between the 2 groups using the 2-sample independent t test or a simple linear regression. Other outcome measures will be analysed in a similar way except if these are skewed when the Wilcoxon rank-sum test will be used. Similarly, a linear mixed models approach will be used to study the 2 study time points (baseline and follow up) in the 2 groups. All tests will be 2 sided and significance will be set at *P* < .05.

## Dissemination Policy

- Patient and Public Involvement (PPI): We secured PPI input from the Pre-sponsorship review panel (PSRP), incorporating their views in the formulation of the study protocol, taking them on-board before ethical clearance and subsequent recruitment of participants. Our PPI rationale is to ensure the presence of older adults with frailty’s voices throughout the study from design onwards, so the research stays meaningful and quality optimised. The PPI advisory group will provide this information throughout the study. The PPI advisory group will optimise our dissemination strategy through online and printed material which will be written for non-specialist audience to assist lay people in understanding the results of the study. We will also seek to publicise the study through printed press and radio interviews. Liaising with KSS- geriatric research network and the BGS, will ensure that the study findings are disseminated. This strategy will help the planning and recruitment of a larger definitive multi-centre study.
- Publications: We will publish the findings in high impact journals. All publications will be published open access or made freely available through the Universities’ website.
- Conferences: We will present the study at the highest impact conferences (eg, British Geriatrics Society annual conference). We will participate in local events, seminars and conferences. Presentations will be offered to interested local older adults’ community groups.

We will ensure that the findings of the pilot study are shared with all study participants who request this information. A study report summarising the main findings will be sent to the participants through their chosen means (either by post or by email), using non-technical language which would make it easy to understand. Participants will be given the opportunity to request further clarification if they do wish to do so after receiving the study report.

## Declaration

All methods used in the study will be carried out in accordance with relevant guidelines and regulations or declaration of Helsinki.

**Ethical approval and consent to participants:** The study has full ethical approval by the NHS Health Research Authority with REC reference number 21/EM/0262 on 14/12/2021. It is being sponsored by the University of Sussex. Written informed consent will be obtained from all study participants and/or their legal guardian(s) for taking part in the study and publication of its findings.

**Consent for publication:** Not applicable.

**Availability of data and materials:** Data sharing is not applicable to this protocol as no datasets have been generated or analysed yet. The datasets generated and/or analysed during the study will be made available on request from the corresponding author.

## References

[1] Fried LP Tangen CM Walston J , et al. Frailty in older adults: evidence for a phenotype. J Gerontol A Biol Sci Med Sci. 2001;56:M146-M156. 10.1093/gerona/56.3.m146 11253156

[2] Office for National Statistics. Living longer: is age 70 the new age 65? Office for National Statistics, May 2019. Accessed March 8, 2020. https://www.ons.gov.uk/peoplepopulationandcommunity/birthsdeathsandmarriages/ageing/articles/livinglongerisage70thenewage65/2019-11-19

[3] British Geriatrics Society. Frailty: what’s it all about? May 2019. Accessed March 8, 2020. https://www.bgs.org.uk/resources/frailty-what’s-it-all-about

[4] Science and Technology Select Committee. House of Lords - ageing: science, technology and healthy living, January 2021. Accessed March 8, 2020. https://publications.parliament.uk/pa/ld5801/ldselect/ldsctech/183/18304.htm#_idTextAnchor004

[5] WHO. WHO Clinical Consortium on Healthy Ageing Topic Focus: Frailty and Intrinsic Capacity, 2017. Accessed March 8, 2020.

[6] Gobbens RJ Luijkx KG Wijnen-Sponselee MT Schols JM . In search of an integral conceptual definition of frailty: opinions of experts. J Am Med Dir Assoc. 2010;11:338-343. 20511101 10.1016/j.jamda.2009.09.015

[7] Siriwardhana DD Hardoon S Rait G Weerasinghe MC Walters KR . Prevalence of frailty and prefrailty among community-dwelling older adults in low-income and middle-income countries: A systematic review and meta-analysis. BMJ Open. 2018;8:e018195. 10.1136/bmjopen-2017-018195 PMC5855322 29496895

[8] Campana EMG Inuzuka S . Vascular aging and arterial stiffness in older adults. Int J Cardiovasc Sci. 2020;33:357-359.

[9] Pereira T Costa T . Determinants of arterial stiffness and vascular aging in the older adult. Int J Cardiovasc Sci. 2020;33(4). doi:10.36660/ijcs.20190068

[10] Aurelian SM Capisizu A Zamfirescu A Cheta D . Arterial stiffness in relation to age and frailty. Atherosclerosis. 2014;235:e225-e226.

[11] Orkaby AR Lunetta KL Sun FJ , et al. Cross-sectional association of frailty and arterial stiffness in community-dwelling older adults: the Framingham heart study. J Gerontol A Biol Sci Med Sci. 2019;74:373-379. 29917058 10.1093/gerona/gly134 PMC6599281

[12] Kannegieter L Tap L Oudshoorn C Van Bruchem-Visser R Mattace-Raso F . Mobility and handgrip strength but not aortic stiffness are associated with frailty in the elderly. J Gerontol Geriatr. 2016;64(1):2-8. http://www.jgerontology-geriatrics.com/article/view/182.

[13] Ochi M Kohara K Tabara Y , et al. Arterial stiffness is associated with low thigh muscle mass in middle-aged to elderly men. Atherosclerosis. 2010;212:327-332. 20554280 10.1016/j.atherosclerosis.2010.05.026

[14] Newman AB Gottdiener JS Mcburnie MA , et al. Associations of subclinical cardiovascular disease with frailty. J Gerontol A Biol Sci Med Sci. 2001;56:M158-M166. 10.1093/gerona/56.3.m158 11253157

[15] Camici GG Savarese G Akhmedov A Lüscher TF . Molecular mechanism of endothelial and vascular aging: implications for cardiovascular disease. Eur Heart J. 2015;36:3392-3403. 26543043 10.1093/eurheartj/ehv587

[16] Ungvari Z Tarantini S Donato AJ Galvan V Csiszar A . Mechanisms of vascular aging. Circ Res. 2018;123:849-867. 30355080 10.1161/CIRCRESAHA.118.311378 PMC6248882

[17] Xu X Wang B Ren C , et al. Recent progress in vascular aging: Mechanisms and its role in age-related diseases. Aging Dis. 2017;8:486-505. 28840062 10.14336/AD.2017.0507 PMC5524810

[18] Ershler WB . A gripping reality: oxidative stress, inflammation, and the pathway to frailty. J Appl Physiol. 2007;103:3-5. 17431088 10.1152/japplphysiol.00375.2007

[19] Saum KU Dieffenbach AK Jansen EHJ , et al. Association between oxidative stress and frailty in an elderly German population: results from the ESTHER cohort study. Gerontology. 2015;61:407-415. 25924722 10.1159/000380881

[20] Álvarez-Satta M Berna-Erro A Carrasco-Garcia E , et al. Relevance of oxidative stress and inflammation in frailty based on human studies and mouse models. Aging. 2020;12:9982-9999. 32461379 10.18632/aging.103295 PMC7288972

[21] Álvarez S Brañas F Sánchez-Conde M Moreno S López-Bernaldo de Quirós JC Muñoz-Fernández MÁ . Frailty, markers of immune activation and oxidative stress in HIV infected elderly. PLoS One. 2020;15:e0230339. 10.1371/journal.pone.0230339 PMC7080240 32187205

[22] Inglés M Gambini J Carnicero JA , et al. Oxidative stress is related to frailty, not to age or sex, in a geriatric population: lipid and protein oxidation as biomarkers of frailty. J Am Geriatr Soc. 2014;62:1324-1328. 24962132 10.1111/jgs.12876

[23] Arauna D García F Rodríguez-Mañas L , et al. Older adults with frailty syndrome present an altered platelet function and an increased level of circulating oxidative stress and mitochondrial dysfunction biomarker GDF-15. Free Radic Biol Med. 2020;149:64-71. 31926293 10.1016/j.freeradbiomed.2020.01.007

[24] Namioka N Hanyu H Hirose D Hatanaka H Sato T Shimizu S . Oxidative stress and inflammation are associated with physical frailty in patients with Alzheimer’s disease. Geriatr Gerontol Int. 2017;17:913-918. 27296166 10.1111/ggi.12804

[25] Tuna Doğrul R Doğan Varan H Kızılarslanoğlu MC , et al. Relationship between frailty and inflammation. Eur J Geriatr Gerontol. 2019;1:17-23.

[26] Marcos-Pérez D Sánchez-Flores M Proietti S , et al. Association of inflammatory mediators with frailty status in older adults: results from a systematic review and meta-analysis. GeroScience. 2020;42:1451-1473. 32803650 10.1007/s11357-020-00247-4 PMC7732924

[27] Puts MT Toubasi S Atkinson E , et al. Interventions to prevent or reduce the level of frailty in community-dwelling older adults: a protocol for a scoping review of the literature and international policies. BMJ Open. 2016;6:e010959. 10.1136/bmjopen-2015-010959 PMC4785293 26936911

[28] Ng TP Feng L Nyunt MS , et al. Nutritional, physical, cognitive, and combination interventions and frailty reversal among older adults: a randomized controlled trial. Am J Med. 2015;128:1225-1236.e1. 10.1016/j.amjmed.2015.06.017 26159634

[29] Bray NW Smart RR Jakobi JM Jones GR . Exercise prescription to reverse frailty. Appl Physiol Nutr Metab. 2016;41:1112-1116. 27649859 10.1139/apnm-2016-0226

[30] Takatori K Matsumoto D . Social factors associated with reversing frailty progression in community-dwelling late-stage elderly people: an observational study. PLoS One. 2021;16:e0247296. 10.1371/journal.pone.0247296 PMC7928521 33657160

[31] Marcucci M Damanti S Germini F , et al. Interventions to prevent, delay or reverse frailty in older people: a journey towards clinical guidelines. BMC Med. 2019;17:193. 31660959 10.1186/s12916-019-1434-2 PMC6819620

[32] British Geriatrics Society. Managing frailty, June 2014. Accessed March 10, 2020. https://www.bgs.org.uk/resources/managing-frailty

[33] Travers J Romero-Ortuno R Bailey J Cooney M-T . Delaying and reversing frailty: a systematic review of primary care interventions. Br J Gen Pract. 2019;69:e61-e69. 10.3399/bjgp18X700241 PMC6301364 30510094

[34] Nunan D . Muscle strength training for reversing frailty: how strong is the evidence? Evid Based Med. 2019;24:199-200. 10.1136/bmjebm-2019-111181 31072921

[35] Mailliez A Guilbaud A Puisieux F Dauchet L . Boulanger Circulating biomarkers characterizing physical frailty: CRP, hemoglobin, albumin, 25OHD and free testosterone as best biomarkers. Results of a meta-analysis. Exp Gerontol. 2020;139:111014. 32599147 10.1016/j.exger.2020.111014

[36] Sánchez-Flores M Marcos-Pérez D Costa S , et al. Oxidative stress, genomic features and DNA repair in frail elderly: a systematic review. Ageing Res Rev. 2017;37:1-15. 28487242 10.1016/j.arr.2017.05.001

[37] Puts MTE Toubasi S Andrew MK , et al. Interventions to prevent or reduce the level of frailty in community-dwelling older adults: a scoping review of the literature and international policies. Age Ageing. 2017;46:383-392. 28064173 10.1093/ageing/afw247 PMC5405756

[38] Asmar R Benetos A Topouchian J , et al. Assessment of arterial distensibility by automatic pulse wave velocity measurement: validation and clinical application studies. Hypertension. 1995;26:485-490. 7649586 10.1161/01.hyp.26.3.485

[39] Ziegelbauer K Schaefer C Steinmetz H Sitzer M Lorenz MW . Clinical usefulness of carotid ultrasound to improve stroke risk assessment: ten-year results from the carotid atherosclerosis progression study (CAPS). Eur J Prev Cardiol. 2013;20:837-843. 22617119 10.1177/2047487312449589

[40] Hooper R . Justifying sample size for a feasibility study, 2019. Accessed March 8, 2020. https://webcache.googleusercontent.com/search?q=cache:KkIoT4-72nsJ:https://www.rds-london.nihr.ac.uk/wpcms/wp-content/uploads/2019/02/Justifying-sample-size-for-feasibility-study-updated-22-Feb-2019.pdf+&cd=3&hl=en&ct=clnk&gl=uk

[41] Billingham SA Whitehead AL Julious SA . An audit of sample sizes for pilot and feasibility trials being undertaken in the United Kingdom registered in the United Kingdom Clinical Research Network database. BMC Med Res Methodol. 2013;13:104. 13. 23961782 10.1186/1471-2288-13-104 PMC3765378

[42] Browne RH . On the use of a pilot. Stat Med. 1995;14:1933-1940. 8532986 10.1002/sim.4780141709

[43] Julious SA . Sample size of 12 per group rule of thumb for a pilot study. Pharm Stat. 2005;4:287-291.

[44] Complior. info center. 2022. Accessed March 12, 2020. http://www.complior.com/info-center

[45] Fukuda Denshi. Development, manufacture and sale of medical equipment. 2022. Accessed March 12, 2020. https://www.fukuda.co.jp/

